# Delayed adrenaline administration prolongs adrenaline-to-ROSC interval in out-of-hospital cardiac arrest

**DOI:** 10.29045/14784726.2025.9.10.2.8

**Published:** 2025-09-01

**Authors:** Michael W. Hubble, Stephen Taylor, Melisa Martin, Sara Houston, Ginny R. Kaplan

**Affiliations:** Wake Technical Community College, USA ORCID iD: https://orcid.org/0000-0002-4683-3767; East Carolina University, USA ORCID iD: https://orcid.org/0000-0002-3824-0283; Methodist University, USA ORCID iD: https://orcid.org/0009-0006-3648-7780; Durham County EMS, USA ORCID iD: https://orcid.org/0009-0005-1570-3854; Methodist University, USA ORCID iD: https://orcid.org/0000-0002-5915-4974

**Keywords:** adrenaline, cardiac arrest, paramedic

## Abstract

**Introduction:**

Previous investigations reveal that protracted resuscitative efforts are associated with poorer long-term patient outcomes. Aside from certain patient characteristics and interventions, such as shockable rhythms, bystander CPR and early defibrillation, little is known about factors influencing resuscitation duration and time to return of spontaneous circulation (ROSC). We hypothesised that early public safety answering point (PSAP) call-receipt-to-pressor (PSAP-to-pressor) administration would decrease the pressor-to-ROSC interval and shorten low-flow duration. Our objective was to quantify the relationship between the PSAP-to-pressor and pressor-to-ROSC intervals.

**Methods:**

We conducted a retrospective analysis using the 2020 ESO dataset containing calls from January to December 2020. Adults with non-traumatic, bystander-witnessed arrests were included. A Cox proportional hazard model was used to determine the association between PSAP-to-pressor interval and pressor-to-ROSC interval while controlling for potential confounders. The end of the event was defined as ROSC, field termination of resuscitation or hospital arrival without ROSC. Patients without ROSC upon hospital arrival were right censored.

**Results:**

Overall, 10,093 patients had data sufficient for analysis. The mean age of the participants was 65.3 (±15.5) years and 64.5% were male. Presumed cardiac aetiology was present in 83.7% of arrests, 29.4% presented with a shockable rhythm and 35.9% attained ROSC. The mean PSAP-to-pressor and pressor-to-ROSC intervals were 16.2 (±5.0) and 14.6 (±11.1) minutes, respectively. The mean time from the first adrenaline administration to the end of the event was 32.7 (±1.0), 41.5 (±1.2) and 51.6 (±3.8) minutes for the 0–10-, 11–20- and 21–30-minute PSAP-to-pressor intervals, respectively (p <0.001). After controlling for confounders, the PSAP-to-pressor time interval was associated with decreased likelihood of ROSC (HR = 0.97 per minute, p <0.001). When stratified by 10-minute increments with 0–10 minutes as reference, PSAP-to-pressor was negatively associated with ROSC for the 11–20- (HR = 0.86, p = 0.002) and 21–30- (HR = 0.66, p <0.001) minute categories.

**Conclusion:**

This retrospective analysis from a national database revealed that increasing delays to first adrenaline administration were associated with prolonged resuscitation duration after drug administration and decreasing likelihood of ROSC.

## Introduction

More than half of coronary heart disease deaths in the United States result from sudden cardiac arrest, yielding roughly 347,000 annual emergency medical services (EMS) responses to out-of-hospital cardiac arrests (OHCA) (Benjamin et al., 2017). Unfortunately, survival is lamentably low, with hospital discharge rates after OHCA languishing at less than 10%, and only 7% surviving with a good neurological outcome (Girotra et al., 2016).

The importance of a witnessed arrest, bystander CPR and early defibrillation on achieving return of spontaneous circulation (ROSC) is well-established (Bækgaard et al., 2017; Horriar et al., 2023; Sasson et al., 2010; Stiell et al., 2004). Additionally, prior investigations have documented the importance of minimising the time interval between collapse and ROSC, as there exists a consistent decline in the likelihood of survival with favourable neurological function as the duration of circulatory no-flow and low-flow states continue unabated (Grunau et al., 2016; Nehme et al., 2016; Reynolds et al., 2013).

In a secondary analysis of the ROC-PRIMED clinical trial data, Reynolds et al. (2016) noted that the likelihood of survival with a modified Rankin Score (mRS) of 0–3 at hospital discharge declined 7% with each minute of CPR duration (OR = 0.93). Similar results were observed in a post hoc analysis of consecutive OHCA patients in British Columbia where the odds of patient discharge with a cerebral performance category of 1–2 declined 9% per minute (OR = 0.91) until ROSC (Grunau et al., 2016). These investigations demonstrate that favourable neurological outcomes are contingent upon rapidly restoring pulses.

For patients who fail to promptly respond to CPR or defibrillation, adrenaline is the first-line pressor of choice in the United States and is administered in an effort to quickly attain ROSC. While the clinical benefits of adrenaline during OHCA continue to be debated, several studies have noted that outcomes are improved when the drug is administered early during the resuscitation timeline (Ran et al., 2020). The exact reasons for this are not fully delineated, but plausible explanations include the myocardium’s increased responsiveness to adrenaline and a less well-established neurological insult during the early phase of resuscitation (Reynolds et al., 2007).

These physiological relationships notwithstanding, earlier administration of adrenaline may also result in a shorter post-drug resuscitation duration, reduced cumulative adrenaline dose and potentially improved neurological outcomes. However, the extent to which the timing of adrenaline administration during the event modifies the time interval between drug administration and ROSC has not been thoroughly investigated. A useful metric for quantifying the timing of pressor administration in cardiac arrest is the interval between call receipt at the call intake centre (also known as the public safety answering point or PSAP) and the time of first drug administration. We hypothesised that faster PSAP-to-pressor administration intervals would decrease the pressor-to-ROSC interval and shorten low-flow duration. Therefore, using a national dataset, we sought to develop a model describing the pressor-to-ROSC interval as a function of the PSAP-to-pressor interval.

## Methods

### Study setting

We conducted a cross-sectional analysis using data from the 2020 ESO Data Collaborative Annual Research data set from ESO, Inc. (Austin, TX). ESO, Inc. is one of the largest providers of EMS electronic health record systems in the United States and includes records from over 2000 US EMS agencies that consented to the release of de-identified data for pre-hospital research. Use of this de-identified dataset was classified as ‘Not Human Subjects Research’ by East Carolina University (UMCIRB 20-002506, 12 April 2024).

### Sample selection

Patients who experienced an OHCA between 1 January and 31 December 2020 were retrospectively identified based on a primary impression of cardiac arrest as recorded by the paramedic. Records meeting our inclusion and exclusion criteria were then extracted for analysis. Adult patients (≥18 years) who experienced a layperson-witnessed, non-traumatic cardiac arrest prior to EMS arrival and who received at least one bolus of adrenaline were included. Patients receiving adrenaline >30 minutes after call receipt were excluded, as prior studies have suggested that initial adrenaline administration >30 minutes post arrest is of questionable effectiveness (Hubble & Tyson, 2017). Patients missing one or more data elements were excluded from the complete case analysis but were included in a sensitivity analysis using imputed data.

### Outcome measure

The principal exposure variable was the PSAP-to-pressor interval, and the primary outcome variable was pre-hospital ROSC.

### Statistical analysis

Using IBM SPSS Statistics version 28 (IBM Corporation), all data were analysed with statistical significance established at p ≤0.05. Continuous variables are reported as means and standard deviations, while categorical variables are reported as percentages. Continuous variables were analysed using Student’s t-test or ANOVA, while categorical data were evaluated using the chi-square test, continuity correction or Fisher’s exact test.

Because resuscitation progresses with the passage of time, we fit a Kaplan-Meier model for post-adrenaline time to ROSC. All patients entered the model upon receipt of the first dose of adrenaline. The likelihood of ROSC, measured as the hazard ratio, was computed for any specified time point during the attempted resuscitation, and patients exited the model (end of event) when they attained ROSC, experienced field termination of resuscitation or remained pulseless with ongoing resuscitation upon hospital arrival. Patients who were transported without ever attaining field ROSC were right censored at the time of hospital arrival. Patients were stratified by PSAP-to-pressor intervals of 0–10, 11–20 and 21–30 minutes. The log rank test assessed the significance of differences across time strata.

 An adjusted analysis used a Cox proportional hazards model to estimate the probability of ROSC throughout the post-adrenaline resuscitation timeline as a function of the PSAP-to-pressor interval, while adjusting for potentially confounding variables. In separate analyses, the PSAP-to-pressor interval was modelled as a categorical variable (0–10, 11–20 and 21–30 minutes), as well as a continuous (per minute) variable. Confounding variables were selected a priori and included patient age, sex and non-Caucasian race; aetiology of arrest; shockable presenting rhythm; bystander CPR; AED shock prior to EMS arrival; and advanced airway placement (blind insertion airway device or endotracheal intubation).

To avoid the bias associated with complete case analysis when a significant number of data points are missing, we imputed the missing data in our data set using the MULTIPLE IMPUTATION command in SPSS. Missing data were imputed under the assumption that data were missing at random. All variables in the Cox regression model were included in the imputation model. We imputed five data sets that were pooled and reanalysed using the same Cox regression model used for the primary analysis. These results were then compared with the complete case analysis as a sensitivity analysis.

## Results

**Figure 1:**
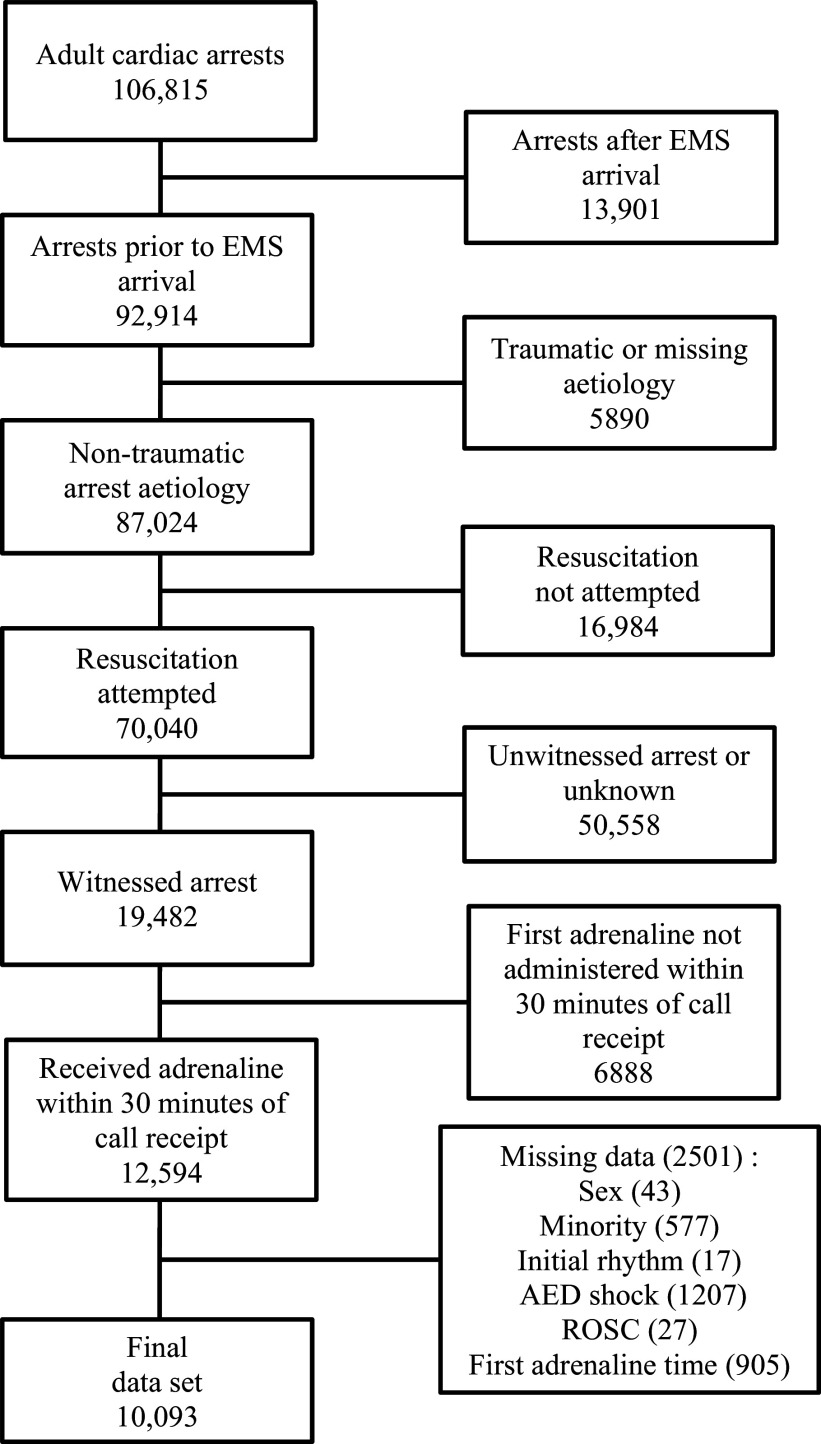
Figure 1. Schematic demonstrating the flow of patients included in the study sample.

During the study period, 106,815 adult patients experienced cardiac arrest, of which 12,594 (11.8%) met inclusionary criteria. A total of 2501 (19.9%) were excluded due to one or more missing data elements, leaving 10,093 (80.1%) patients in the final data set for complete case analysis (Figure 1). In the complete case analysis, males accounted for 64.5% of the sample, while non-Caucasian races represented 26.1%. The mean age of participants was 65.3 (±15.5) years. The majority (83.7%) of arrests were of presumed cardiac aetiology, and 29.4% presented in a shockable rhythm. The mean EMS response time was 7.7 (±3.7) minutes, and bystander CPR was performed in 30.0% of cases. In 5.1% of patients, one or more AED defibrillations were attempted prior to the arrival of EMS. An advanced airway was successfully placed in 8658 (85.8%) patients. The mean PSAP-to-pressor interval was 16.2 (±5.1) minutes. Overall, 3626 (35.9 %) experienced ROSC prior to hospital arrival.

Compared to patients without ROSC, patients attaining ROSC were slightly younger and more likely to present with a shockable rhythm, receive bystander CPR, be defibrillated with an AED and receive the first dose of adrenaline much sooner (8.4 (±5.1) minutes versus 15.6 (±4.9), p <0.001). Additional details of the sample are provided in Table 1.

**Table 1.** Univariate comparison of those with and without ROSC among patients with complete data.

Variable	All patients (n = 10,093)	Without ROSC (n = 6467)	With ROSC (n = 3626)	p-value
Age (years)	65.33 (± 15.58)	65.56 (± 15.50)	64.91(± 15.71)	0.045
Male sex (%)	64.5%	66.7%	60.5%	<0.001
Non-Caucasian race (%)	26.1%	26.7%	24.9%	0.047
Aetiology of arrest: Presumed cardiac (%) Respiratory (%) Drug overdose (%) Other (%)	83.7% 11.2% 2.1% 3.0%	85.5% 9.8% 1.8% 3.0%	80.5% 13.7% 2.7% 3.2%	<0.001
Received bystander CPR (%)	30.0%	29.1%	31.7%	0.006
Initial shockable rhythm (%)	29.4%	27.4%	33.0%	<0.001
Received AED shock prior to EMS arrival (%)	5.1%	4.8%	5.8%	0.024
Received advanced airway placement (%)	85.8%	84.4%	88.2%	<0.001
EMS response time (minutes, ±SD)	7.72 (± 3.78)	7.83 (± 3.86)	7.51 (± 3.62)	<0.001
PSAP call receipt to first adrenaline interval (minutes, ±SD)	16.60 (± 5.16)	15.66 (± 4.91)	8.49 (± 5.1)	<0.001
PSAP call receipt to ROSC interval (minutes, ±SD)	30.73 (± 12.51)	N/A	N/A	N/A
ROSC (%)	35.9%	N/A	N/A	N/A

AED = automated external defibrillator; CPR = cardiopulmonary resuscitation; EMS = emergency medical services; PSAP = public safety access point; ROSC = return of spontaneous circulation; SD = standard deviation.

The Kaplan-Meier analysis revealed statistically significant differences in the time to end of event (ROSC or censoring) stratified by PSAP-to-pressor interval. The mean time from the first adrenaline administration to the end of the event was 32.7 (±1.0), 41.5 (±1.2) and 51.6 (±3.8) minutes for the 0–10-, 11–20- and 21–30-minute PSAP-to-pressor intervals, respectively (p <0.001).

**Figure 2:**
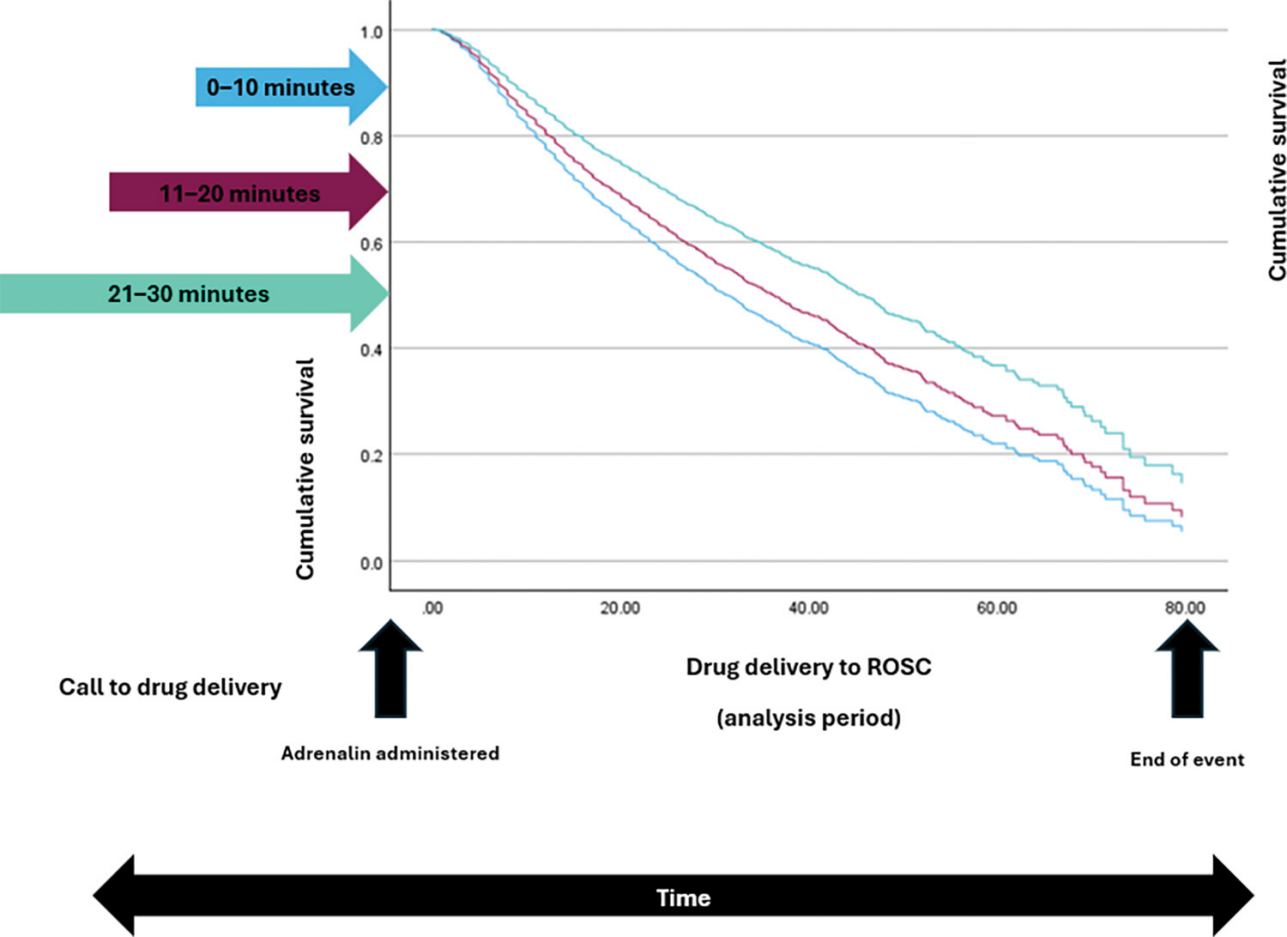
Figure 2. Adjusted survival function for PSAP-to-pressor intervals.

The proportional hazard assumption was confirmed via log-log and Schoenfeld residual plots; therefore, a Cox proportional hazard model was used to evaluate the influence of the PSAP-to-pressor interval on the time to ROSC, while controlling for potentially confounding variables. Results of the Cox model treating the PSAP-to-pressor interval as a categorical variable are presented in both Table 2 and Figure 2. When stratified by 10-minute increments with 0–10 minutes as reference, increasing PSAP-to-pressor intervals were negatively associated with the likelihood of ROSC (HR = 0.86, p = 0.002 and HR = 0.66, p <0.001 for the 11–20- and 21–30-minute categories, respectively). Male sex and non-Caucasian race were also associated with a significantly decreased likelihood of ROSC. Conversely, patients with an initially shockable rhythm were at increased likelihood of obtaining ROSC. Additionally, patients with either respiratory or drug overdose aetiologies of arrest were more likely to experience ROSC compared to patients with a presumed cardiac aetiology. Similar results were observed when the PSAP-to-pressor interval was modelled as a continuous variable. Notably, the risk of ROSC declined 3% per minute (HR = 0.970, p <0.0.001).

**Table 2.** Adjusted hazard ratio for ROSC.

	Complete case analysis (n = 10,093)			Imputed data analysis (n = 12,594)		
Variable	Hazard ratio	95% CI	p-value	Hazard ratio	95% CI	p-value
Age (per year)	1.001	0.999–1.003	0.278	1.001	0.999–1.003	0.271
Male sex	0.746	0.698–0.799	<0.001	0.762	0.717–0.811	<0.001
Non-Caucasian race	0.925	0.857–0.999	0.046	0.925	0.859–0.995	0.037
Aetiology of arrest: Presumed cardiac Respiratory Drug overdose Other	reference 1.449 1.616 1.181	1.315–1.597 1.309–1.994 0.979–1.424	<0.001 <0.001 0.081	reference 1.476 1.554 1.116	1.352–1.612 1.288–1.875 0.942–1.323	<0.001 <0.001 0.204
Received bystander CPR	1.039	0.968–1.115	0.293	1.045	0.979–1.115	0.188
Initial shockable rhythm	1.184	1.099–1.275	<0.001	1.209	1.128–1.295	<0.001
Received AED shock prior to EMS arrival	1.052	0.911–1.215	0.488	1.046	0.908–1.206	0.530
Received advanced airway placement	0.996	0.900–1.102	0.935	1.020	0.932–1.115	0.672
Call received to first adrenaline 0–10 minutes 11–20 minutes 21–30 minutes	reference 0.860 0.663	0.780–0.948 0.589–0.747	0.002 <0.001	reference 0.874 0.690	0.798–0.956 0.619–0.769	0.003 <0.001

AED = automated external defibrillator; CI = confidence interval; CPR = cardiopulmonary resuscitation; EMS = emergency medical services.

**Figure 3:**
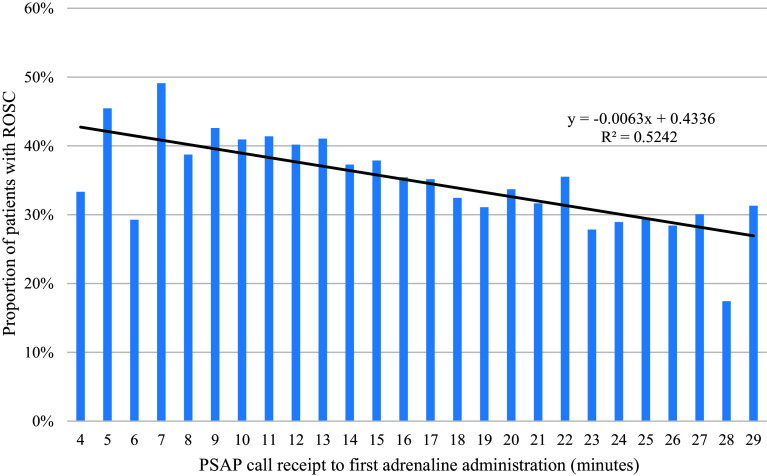
Figure 3. Proportion of patients attaining ROSC by PSAP-to-pressor interval.

With respect to the proportion of patients attaining ROSC, there was a significant negative trend with increasing PSAP-to-pressor intervals (Figure 3).

We also performed a sensitivity analysis using multiple imputation to minimise the risk of bias emanating from missing data. Applying the same Cox model to the imputed data, the ROSC risk decreased similarly (HR = 0.87, p = 0.003 and HR = 0.69, p <0.001) for the 11–20- and 21–30-minute categories, respectively. Overall, we found no meaningful differences between the results of the original and imputed data sets.

## Discussion

For most victims of OHCA, survival predominantly hinges on expeditiously achieving field ROSC; whereas transporting pulseless patients to the hospital for continued resuscitation yields few additional survivors (Stub et al., 2014; Wampler et al., 2012). In a large national database, we observed a negative association between delays in adrenaline administration and the likelihood of field ROSC. In this time-to-event analysis, escalating intervals between call receipt and first adrenaline administration were associated with decreasing probabilities of ROSC, and equally as important, increasing time to ROSC after drug administration. This association remained significant even after adjusting for other important patient-, scene- and treatment-level factors. Prior studies have correlated delayed adrenaline administration with poor outcomes and notably lower rates of ROSC, but have not explicitly evaluated the influence of these delays on post drug-delivery CPR duration and delays in attaining ROSC (Okubo et al., 2021; Ran et al., 2020; Sakamoto et al., 2025).

The importance of the current findings is that prolonged resuscitation duration is associated with poor neurological function, and a more rapid recovery of pulses is a key step towards meaningful outcomes. Previous reports have demonstrated that a shorter time to ROSC is independently associated with both survival and favourable neurological outcomes. In a single-centre study, Reynolds et al. (2013) observed that survival to discharge with favourable neurological outcomes (mRS 0–3) declined precipitously with each additional minute of EMS-provided CPR duration (OR = 0.84, p = 0.004). Of those discharged with mRS 0–3, 90% had attained ROSC within 16 minutes of the initiation of CPR. Similarly, in a post-hoc analysis of consecutive OHCA patients in British Columbia, Grunau et al. (2016) reported that the odds of patient discharge with a cerebral performance category of 1–2 declined 9% per minute (OR = 0.91) until ROSC was attained. A secondary analysis of the ROC-PRIMED clinical trial data found that the likelihood of survival with an mRS score of 0–3 at hospital discharge declined 7% with each minute of CPR duration (OR = 0.93, p <0.001) (Reynolds et al., 2016). Although they lacked data on neurological outcomes, Nehme et al. (2016) reported a 13% reduction in the odds of survival to hospital discharge for every minute increase in CPR duration (OR = 0.87, p <0.01). In their evaluation of patients presenting with ventricular fibrillation (VF) in Funabashi City, Japan, Arima et al. (2015) reported both a declining 24-hour survival rate and a poorer 30-day neurological outcome with increasing pre-hospital CPR duration. They noted that the cut-off values for survival and favourable neurological outcome were 27 minutes and 17 minutes CPR duration, respectively. Significantly, among patients who survived OHCA and were admitted to an ICU in Tokyo, Komatsu et al. (2014) identified the time interval between collapse and ROSC as the only independent predictor of favourable neurological outcome (OR = 0.86, p <0.001) among a collection of pre-hospital and post-admission factors.

The aforementioned studies demonstrate that favourable neurological outcome is profoundly more sensitive to delays in attaining ROSC than is short-term survival. In addition to the underlying and progressive detrimental effects of myocardial and neuronal ischaemia, the physiological basis for poor outcomes may also lie in the time-varying pharmacological effects of adrenaline itself. In a superbly designed animal experiment, Choi et al. (2024) evaluated the influence of adrenaline on cerebral perfusion pressure (CePP), cerebral blood flow (CBF) and coronary perfusion pressure (CoPP). In their experiment, 24 swine were randomly assigned to early, intermediate and late adrenaline administration. VF was induced and remained untreated for four minutes, at which point mechanical CPR was initiated. A 1 mg intravenous bolus of adrenaline was then administered at five minutes (early), 10 minutes (intermediate) or 15 minutes (late) after the onset of VF. In comparison to the intermediate and late groups, early adrenaline administration was associated with higher CePP, CBF and CoPP. Moreover, adrenaline showed a limited increase in the intermediate group and even a negative effect in the late group on CePP and CBF. These findings are similar to those of some animal studies reporting initially positive, but subsequently waning, beneficial effects with successive adrenaline doses administered later in the resuscitation timeline (Mavroudis et al., 2020; Nosrati et al., 2019), although others have reported conflicting results (Ristagno et al., 2007, 2009). Interestingly, the timing of late administration in the Choi et al. (2024) study (15 minutes post arrest) reflects the typical timing of the first drug administration in many EMS systems (Cantrell et al., 2013; Ewy et al., 2015; Hubble & Tyson, 2017).

Given the decreasing effectiveness of adrenaline to shorten CPR duration as arrest time progresses, coupled with the drug’s ostensibly time-dependent effects on CePP and CBF, and presumably neurological outcome, efforts should focus on devising means for faster drug delivery for patients who do not respond to CPR and defibrillation. Unfortunately, such a goal is likely to be challenging. For most OHCA patients, much of the delay in drug delivery is attributable to the EMS response interval, which offers limited opportunities for meaningful improvement in most EMS systems. Consequently, the only option within the context of existing EMS system designs is modifying on-scene care, which might include prioritising drug delivery over intubation, and administering adrenaline via the IO or IM routes, rather than the presumably slower to establish IV route. Although these strategies may be easy to implement, they are unlikely to produce little more than modest reductions in the PSAP-to-pressor interval (Hubble & Tyson, 2017). In addition, recent randomised controlled trials have failed to demonstrate superiority of the IO route over the IV route (Couper et al., 2024; Vallentin et al., 2024).

Ultimately, more consequential improvements in OHCA outcomes among patients requiring adrenaline may require innovating a model of care that, when feasible and without interfering with chest compressions and defibrillation, allows first responders to administer the first dose of adrenaline because they often arrive to the scene prior to EMS. With adequate additional training and medical oversight, first responders could establish IO access and administer the first bolus of adrenaline or administer intramuscular (IM) adrenaline, which is a skill already on the formulary in many American states. As well, other avenues of reducing the PSAP-to-pressor interval may hold promise. Experimental animal models suggest that IM adrenaline administered early may be as effective as early-administered IV adrenaline and is superior to delayed IV administration (Mauch et al., 2013). Additionally, in a field feasibility study by Pugh et al. (2021), investigators observed a three-minute reduction in the PSAP-to-pressor interval when the first dose of adrenaline was administered IM by paramedics. Although their sample was underpowered to detect a statistically significant difference when compared to historical controls, rates of survival to hospital discharge were similar between the groups (10%, 8% and 7% for IM, IV and IO, respectively). Palatinus et al. (2024), using a before and after study design, observed that an initial IM dose of adrenaline in addition to standard care was associated with improved survival to hospital admission, survival to hospital discharge and functional survival. Should follow-up randomised trials confirm such a survival advantage, an IM adrenaline procedure could be extended to first responders for drug administration prior to EMS arrival. As well, one could foresee an extension of pre-EMS adrenaline administration via an autoinjector by laypersons. Adrenaline autoinjectors of appropriate doses could be placed alongside automated external defibrillators for use by witnesses to OHCA. Obviously, introducing such programmes would require significant planning, funding, medical oversight, changes to existing laws regarding administration by non-healthcare providers and post-implementation evaluation. Nonetheless, if IM adrenaline proves to be effective in OHCA and first responder and bystander IM adrenaline programmes were to be successfully implemented, it may be possible to substantially reduce the PSAP-to-pressor interval and potentially improve outcomes for some patients.

### Limitations

Like many studies of retrospective design, this study is subject to several limitations, including the completeness and accuracy of data reporting. The study design also did not permit implying causality or making recommendations on whether vasopressors should or should not be given. Instead, the aim was to clarify whether there was a time-dependent relationship between the PSAP-to-pressor and pressor-to-ROSC intervals.

Our data set suffered from a large number of patients with missing data points. To minimise this impact, missing data were imputed and analysed as a sensitivity analysis. The results of this analysis suggest that the missing data did not substantially affect our point estimates or conclusions.

Because our methods incorporated a time-to-event analysis, we cannot rule out the potential for the unmeasured influence of varying termination of resuscitation (TOR) guidelines across EMS agencies. Some patients with field TOR may ultimately have attained ROSC had resuscitative efforts persisted. Consequently, we cannot eliminate TOR guidelines, or the lack thereof, as a source of unmeasured confounding.

Our data were collected during the COVID-19 pandemic, which may have influenced our results. It is unclear how the pandemic may have impacted resuscitation, decisions regarding field TOR or patient outcomes.

Finally, hospital discharge data were not universally available for this study, and the influence of post-vasopressor duration of resuscitation on longer-term outcomes remains unknown. However, we believe that early ROSC is an essential first step towards neurological recovery. As such, our findings should encourage further investigation of the intertwined relationships between the timing of adrenaline administration, early restoration of spontaneous circulation and neurological salvage.

## Conclusion

Within the limits of our retrospective study design using a national database, we found that increasing delays to first adrenaline administration were associated with prolonged resuscitation duration following drug administration and a decreasing likelihood of ROSC. Thus, the effectiveness of adrenaline in fostering ROSC is not constant and appears to vary with respect to when adrenaline is administered in the resuscitation timeline.

This analysis contributes to the exploration and comprehension of the relationship between the PSAP-to-pressor and pressor-to-ROSC intervals. Additional study is needed to further explicate not only this relationship but also the contribution towards positive neurological outcomes.

## Author contributions

Each author contributed to the design of the study. MH conducted the statistical analysis and drafted the manuscript, and all authors contributed substantially to its revision. MH acts as the guarantor for this article.

## Conflict of interest

The authors report no conflict of interest. This manuscript was not prepared in collaboration with investigators from any entity. The data were provided by ESO, Inc. and the authors wish to express their appreciation to ESO for their assistance. The content derived from this data set remains the property of ESO, Inc. ESO is not responsible for any claims arising from works based on the original data, text, tables or figures. The authors alone are responsible for the content and writing of the article.

## Ethics

Use of this de-identified dataset was classified as ‘Not Human Subjects Research’ by East Carolina University Institutional Review Board (UMCIRB 20-002506, 12 April 2024).

## Funding

None.

## Statement of generative AI in scientific writing

The authors did not use a generative artificial intelligence (AI) tool or service to assist with preparation or editing of this work.
